# Assessing the feasibility of eHealth and mHealth: a systematic review and analysis of initiatives implemented in Kenya

**DOI:** 10.1186/s13104-017-2416-0

**Published:** 2017-02-10

**Authors:** Martin Njoroge, Dejan Zurovac, Esther A. A. Ogara, Jane Chuma, Doris Kirigia

**Affiliations:** 1 Department of Public Health Research, KEMRI-Wellcome Trust Research Programme, PO BOX 43640-00100, Nairobi, Kenya; 2 Centre for Tropical Medicine, Nuffield Department of Clinical Medicine, University of Oxford, CCVTM, Oxford, OX3 7LJ UK; 3 Center for Global Health and Development, Boston University School of Public Health, 85 East Concord Street, Boston, MA 02118 USA; 4 Ministry of Health, Division of eHealth, Nairobi, Kenya

**Keywords:** Kenya, eHealth, mHealth, Telemedicine, Health information systems, Evaluation, Health equity

## Abstract

**Background:**

The growth of Information and Communication Technology in Kenya has facilitated implementation of a large number of eHealth projects in a bid to cost-effectively address health and health system challenges. This systematic review aims to provide a situational analysis of eHealth initiatives being implemented in Kenya, including an assessment of the areas of focus and geographic distribution of the health projects. The search strategy involved peer and non-peer reviewed sources of relevant information relating to projects under implementation in Kenya. The projects were examined based on strategic area of implementation, health purpose and focus, geographic location, evaluation status and thematic area.

**Results:**

A total of 114 citations comprising 69 eHealth projects fulfilled the inclusion criteria. The eHealth projects included 47 mHealth projects, 9 health information system projects, 8 eLearning projects and 5 telemedicine projects. In terms of projects geographical distribution, 24 were executed in Nairobi whilst 15 were designed to have a national coverage but only 3 were scaled up. In terms of health focus, 19 projects were mainly on primary care, 17 on HIV/AIDS and 11 on maternal and child health (MNCH). Only 8 projects were rigorously evaluated under randomized control trials.

**Conclusion:**

This review discovered that there is a myriad of eHealth projects being implemented in Kenya, mainly in the mHealth strategic area and focusing mostly on primary care and HIV/AIDs. Based on our analysis, most of the projects were rarely evaluated. In addition, few projects are implemented in marginalised areas and least urbanized counties with more health care needs, notwithstanding the fact that adoption of information and communication technology should aim to improve health equity (i.e. improve access to health care particularly in remote parts of the country in order to reduce geographical inequities) and contribute to overall health systems strengthening.

**Electronic supplementary material:**

The online version of this article (doi:10.1186/s13104-017-2416-0) contains supplementary material, which is available to authorized users.

## Background

eHealth is the use of information and communication technologies (ICT) for health as defined by the World Health Organization (WHO) [1]. The adoption of ICT in health is increasingly being implemented in sub-Saharan Africa, particularly in Kenya with an aim to strengthen different components of the health system. Adoption of eHealth promises a number of potential benefits to the health system that include increased efficiency in health care, improvement in quality of care, costs reduction, enhanced health system governance structures, thus extending the provision of healthcare beyond its conventional boundaries [2].

In addition Kirigia et al. noted that, ICT treats geographically displaced resources as if they were centralized thus promoting economies of scale [3]. Embracing eHealth can indeed facilitate equitable distribution of healthcare to the marginalised areas and vulnerable population groups. Due to the numerous potential benefits arising from the adoption of eHealth in health systems, the private and public sectors have increased investment in eHealth interventions in low and middle income countries (LMICs) [4]. However, several challenges hinder successful implementation of eHealth interventions in LMICs. These include inadequate infrastructure and equipment; insufficient human resources and skills; inadequate legislation; insufficient or unreliable power supply; scarce funding for sustainability; lack of government buy-in and ownership and weak evaluation mechanisms leading to the implementation of a myriad of pilot projects that are rarely scaled up [5].

In Kenya, the Government Vision 2030 policy identifies ICT as a key determinant of the attainment of an economic pillar relating to business process offshoring (outsourcing). This implies that young people can provide business services via the internet to offshore companies and organisations in the developed world in an effort to promote Kenya as the top business destination in Africa [6]. Due to this, the country has experienced tremendous ICT sector growth particularly in telecommunications, software development and software as a service, which is the delivery of applications to the end user via the web. For example, the increased investment by the private and public sectors [7] in developing a national fibre optic infrastructure and utilisation of mobile devices [8, 9] has enhanced acceptance of ICT leading to economic growth in Kenya [10]. In 2011, it was estimated that 93% of Kenyan households owned a mobile phone [8] with at least two-thirds of the entire population having access to mobile money. Indeed, Kenya is leading in ICT growth in East Africa with the capital Nairobi referred to as a “Silicon Savannah” due to its role as an information technology hub [11]. In spite of the ICT growth in Kenya, evidence shows that mobile phone ownership and usage is associated with gender, level of education, literacy, urbanization and the socio-economic status of the individuals [12, 13].

The development of ICT sector in Kenya coupled with presence of a sizable ICT human resource contributed to increased implementation of eHealth projects in the country. In support of the growing eHealth projects in Kenya, the Ministry of Health in 2011 launched the first Kenya National eHealth Strategy 2011–2017 [14]. According to the strategy, the Kenyan health system faces the challenge of rising cost and demand for quality health care services against a backdrop of skilled health personnel shortages. Hence, the eHealth strategy aimed to address these challenges by harnessing ICT for improved healthcare delivery and health system strengthening. The national eHealth strategy outlined five specific strategic areas of focus to aid eHealth project implementation in the country. These include telemedicine, health information systems (including electronic health records), mHealth (health through the use of mobile devices), eLearning (including distance education or learning) and health information for citizens (health information provision to the patients). The strategy also anticipated that ICT would promote and deliver efficient healthcare services to Kenyans and consumers beyond Kenya’s borders [14].

Since the launch of the national strategy, the public and private sectors have been implementing small and large scale eHealth innovations encompassing all five strategic areas [9, 11, 15–21]. However, there is dearth of evidence of initiatives being implemented in the country. Specifically, there is lack of a registry of all projects under implementation and documentation of their specific characteristics including contributions to strengthening the Kenyan health system. This review therefore, provides an inventory of all eHealth initiatives in Kenya by their area of implementation, project characteristics and evaluation status. The evidence from this analysis will provide knowledge and better understanding of the eHealth activities implemented in Kenya to date, and their impacts in strengthening the overall health system. This research output is part of a broader ongoing mixed-methods study that aims to evaluate the impact of eHealth in terms of strengthening the Kenyan health system through reducing health inequities and promoting good governance.

## Methods

### Search strategy

A search of peer reviewed and non-peer reviewed sources of relevant information was performed. The search for peer reviewed literature was conducted in PubMed, Embase, Web of Science, Econlit, SocIndex, Toc Premier, Cochrane Database of Systematic Reviews, INASP, LISTA, CAB Abstracts, Directory of Open Access Journals, EBSCOhost (incorporating Academic Search Complete) and Google Scholar. This however was not sufficient as there were large number of eHealth projects in Kenya that were not reported in peer reviewed literature. Therefore, to ensure all relevant information was captured, a search was extended to non-peer reviewed sources including web-based portals for eHealth, profit and not-for-profit organizational websites, newspaper articles, and blogs. The search was also extended to eHealth implementers who had not yet published information, government documents and organizations reports such as those of WHO, m-Health alliance and International Development Research Centre (IDRC) among others.

The first search strategy was performed on all documents related to eHealth projects under implementation in Kenya in peer reviewed sources. The search terms used with AND/OR included;

- Kenya, mhealth AND/OR m-health, ehealth AND/OR e-health, mobile, mobile phone, cellular phone, cell phone, electronic health record, electronic health*, internet, text messag*, SMS, telemed*, e-learning, evaluation.

These search terms and key words were used so as to capture all the relevant projects in Kenya. Since eHealth is the use of use of information and communication technologies (ICT) for health [1], the key words were selected so as to address all strategic areas of implementation. The strategic areas included telemedicine, health information systems (including electronic health records), mHealth (health through the use of mobile devices) and eLearning (including distance education or learning) as defined in the Kenya National eHealth Strategy) [14]. All strategic areas of eHealth implementation were included in order to develop a comprehensive registry of projects considering this was the first situational analysis in Kenya.

For the non-peer reviewed online databases, a similar search strategy using the same keywords and concepts as in the peer reviewed strategy was used as shown in Table 1. A search for relevant documents on web-portals was also undertaken including profit and not-for-profit organizations websites and blogs documenting eHealth projects in Kenya consisting of 3–5 steps as illustrated in Table 1. The next step, which can be deemed as subjective, was initiated through our research network in Kenya where personal communication with some eHealth implementers was sought to gather information relating to their innovation. Finally, hand searches of cited references in documents and reports obtained were finally used to augment the search. Opinion pieces, publications and letters to the editor lacking relevant data were excluded. The search strategy consisted of seven steps illustrated in Table 1 and involved eHealth initiatives being implemented in Kenya.

**Table 1:** Search strategy used (Source: authors’ synthesis)

Step	Description
1	Peer reviewed sources of information: MEDLINE, Embase, Web of Science, Econlit, SocIndex, Toc Premier, Cochrane Database of Systematic Reviews, INASP, LISTA, EBSCOhost, Directory of Open Access Journals, Google Scholar
2	Non-peer reviewed sources of information: Africa-wide information, newspapers(Nation and Standard), organizational reports (WHO, m-health alliance, IDRC)
3	Web-portals for eHealth projects in Kenya
4	Profit and not- for-profit organizational websites
5	Blogs and other networks
6	Personal communication with implementers
7	Hand searches of references in included documents

Since, the ICT sector and the adoption of eHealth initiatives in Kenya is fairly nascent [8], the time period was not specified. All publications selected and information included in this study had to have been implemented in Kenya with clear details of the specific eHealth application under implementation. The retrieved records from each search were screened for eligibility. Conflicting opinions were resolved through discussion amongst the authors.

### Data extraction

The description of data extracted from the eligible publications involved description of eHealth application; strategic area of project implementation (either mHealth, telemedicine, health information systems or eLearning as defined in the Kenya National eHealth Strategy) [14]; and geographic location of the project. Other descriptive criteria included thematic area or domain of the project as informed by previous research undertaken by Kallander et al. [22] and Labrique et al. [23]; project implementation period; specific health focus and evaluation status. The data on the funding source was also extracted where applicable. In instances where necessary data was not clearly stated, communication was initiated with the relevant publication authors to seek clarification and if possible share missing data. However, due to the sensitive nature of this information, most private projects were not prepared to disclose the information deemed as delicate.

### Categorization of eHealth projects in Kenya

In light of the vast number of projects identified, efforts were made to classify the specific areas of eHealth project implementation through development of different categories. The categories facilitated assessment of the extent to which eHealth projects focused on specific facets of eHealth at the expense of others. The categorization consisted of four parts. First was the strategic area; second, the thematic area; third, the health area of focus; and finally the geographic location of all the projects being implemented.

The strategic areas of project implementation were adopted from the Kenya National eHealth Strategy 2011–2017 [14] namely, telemedicine, health information systems, mHealth and eLearning. Due to overlap with other areas, the strategic area referring to health information for citizens was not analysed separately but included in other project implementation areas.

The second categorization centred on thematic areas was adopted from prior research work that highlighted the most common applications in mHealth and eHealth [22, 23]. The twelve most common applications of mHealth as described by Labrique et al. [23] provides a robust framework that was applied in this study to categorize eHealth projects in Kenya. These includes: (1) client education and behaviour change communication; (2) sensors and point-of-care diagnostics; (3) registries and vital event tracking; (4) data collection and reporting; (5) electronic health records; (6) electronic decision support such as information, protocols, algorithms, checklists; (7) provider-to-provider communication such as user groups, consultations; (8) provider work planning and scheduling; (9) provider training and education; (10) human resource management; (11) supply chain management; and (12) financial transactions and incentives (i.e. use of mobile money transfers and banking services to pay for health services and incentivise patients).

The health focus category entailed the specific health areas of projects focus including malaria, HIV/AIDS, tuberculosis, primary care, health care financing among others. Whilst the project geographic location category adopted, details the specific setting in which the project was executed. Most eHealth projects were designed to be implemented in specific geographical locations with a few projects implemented nationally.

This study produced geo-coded maps and plotted all identified projects based on their strategic area of implementation. The maps were layered based on urbanization illustrating the number of residents living in towns with at least 2000 inhabitants [24] and marginalised counties as identified by the Commission on Revenue Allocation (CRA) [25]. The marginalised counties were identified based on the County Development Index (CDI) developed by the CRA which is a composite index consisting of indicators that measures the state of health (for example, health, education and infrastructure are all weighted at 28%, and the level of poverty in a county indicator at 16%) [25, 26]. Based on these different criteria, the following fourteen counties are classified as marginalised with CDI ranging from 0.27 to 0.52. The counties are Turkana, Mandera, Wajir, Marsabit, Samburu, West Pokot, Tana River, Narok, Kwale, Garissa, Kilifi, Taita Taveta, Isiolo and Lamu.

## Results

**Fig. 1:**
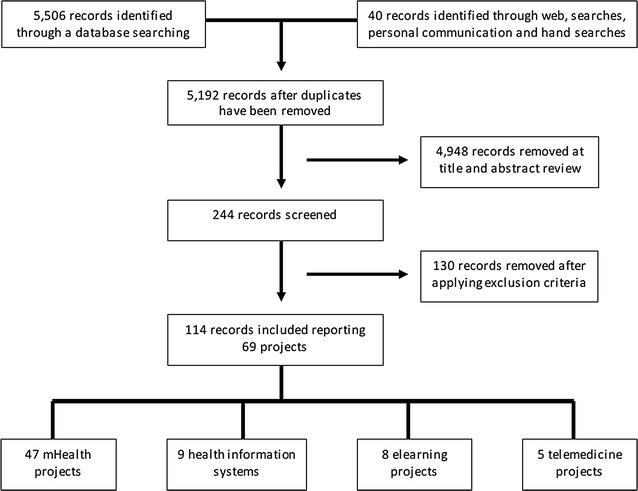
Search results. *Source*: authors’ synthesis

The search strategy identified 5506 citations through the peer reviewed and non-peer reviewed sources of information (step 1 and 2 in Table 1). An additional 40 publications were retrieved through hand searches of organisations websites, relevant documents, and blogs from web searches. 354 records were removed since they were duplicates. A further 4948 publications that did not meet the criteria were excluded during the screening of titles and abstracts. Those that were excluded had not described the eHealth project clearly, and/or were not implemented in Kenya or lacked the relevant information. Of the remaining 244 records fully screened, 130 were excluded after applying exclusion criteria. Of these, 86 were not relevant, 2 were correspondence letters, 3 lacked intervention of interest, 3 were opinion pieces, 4 were reviews and 32 were not executed in Kenya. The search concluded with 114 citations reporting 69 eHealth projects (Fig. 1).

The attached Additional file 1 outlines the categorisation of the sixty-nine eHealth projects according to the geographic location (county), strategic area of implementation, project period, thematic area, health focus, description of eHealth application and evaluation status of the project under implementation.

### Geographic location of the projects by urbanization and marginalisation

**Fig. 2:**
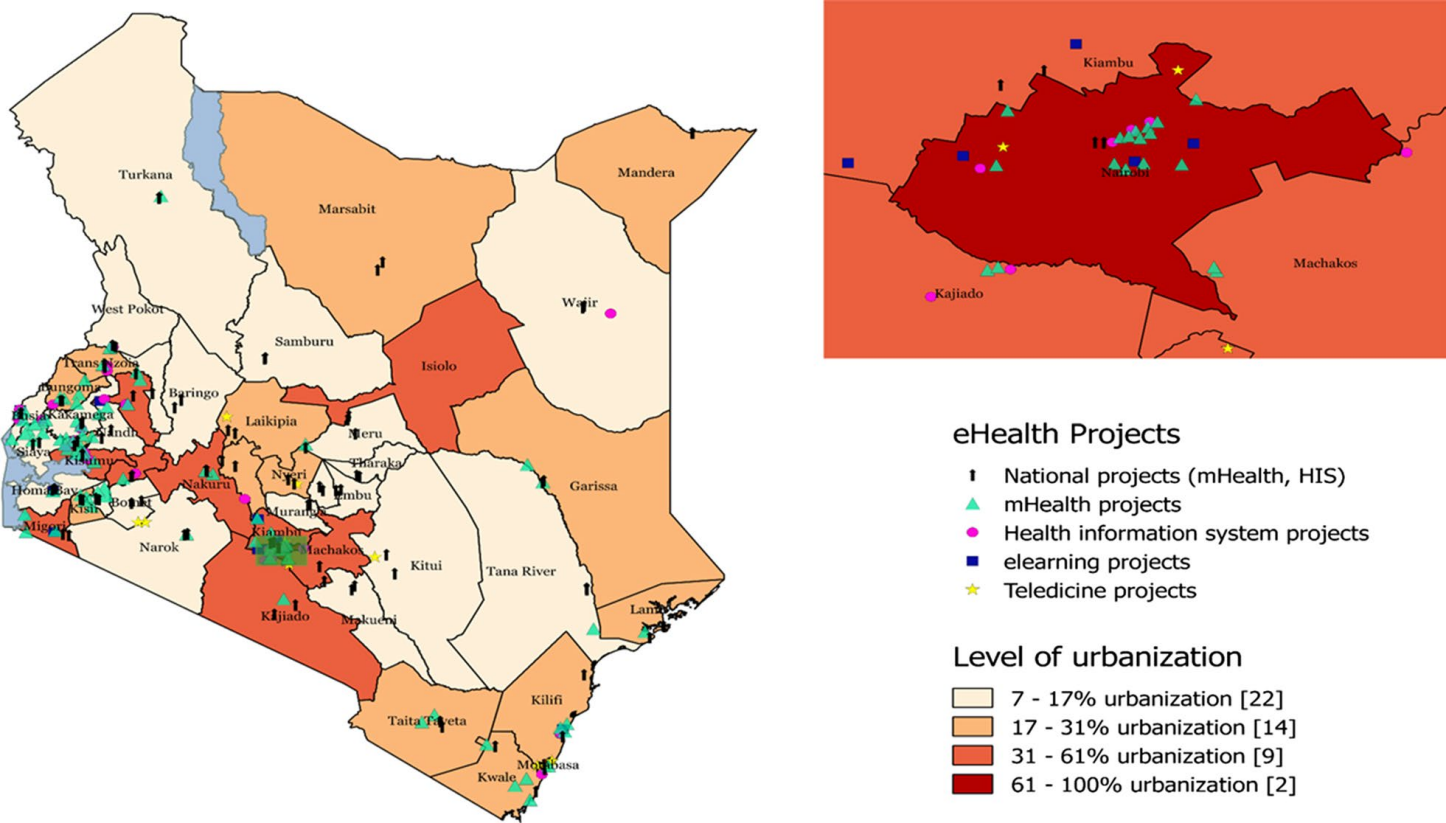
Distribution of eHealth projects in Kenya by urbanization. Map A consisting of all the eHealth projects under implementation in 47 counties in Kenya. Map shows geographical locations of all eHealth initiatives categorised by strategic area of implementation and national projects. Map background is divided according to percentage of urbanization (see *colour bars*) and the number of counties in *square brackets*. Map B (*Inset*) Nairobi County (capital city) and neighbouring counties. *Source*: authors’ synthesis. No data permission was required

Thirty-five out of the 47 counties in Kenya had at least one eHealth project being implemented excluding the national projects which were executed in health centres. Additionally, most of the projects (n = 41) were implemented in multiple counties thus the total number of mapped projects excluding national projects (n = 148) were greater than the number of actual projects (n = 69). The capital city Nairobi had the most number (n = 24) of mapped projects. In other large urban centres such as Kisumu (n = 13) and Busia (n = 9) the eHealth projects were also common. The least urbanised counties such as Turkana (n = 1), Wajir (n = 1) and Garissa (n = 2) had the least number of eHealth projects. However, the other least urbanised counties such as Samburu, Marsabit and Mandera had no eHealth project being implemented. In total there were a total of eleven projects being executed in the least urbanized counties as demonstrated by Fig. 2.

**Fig. 3:**
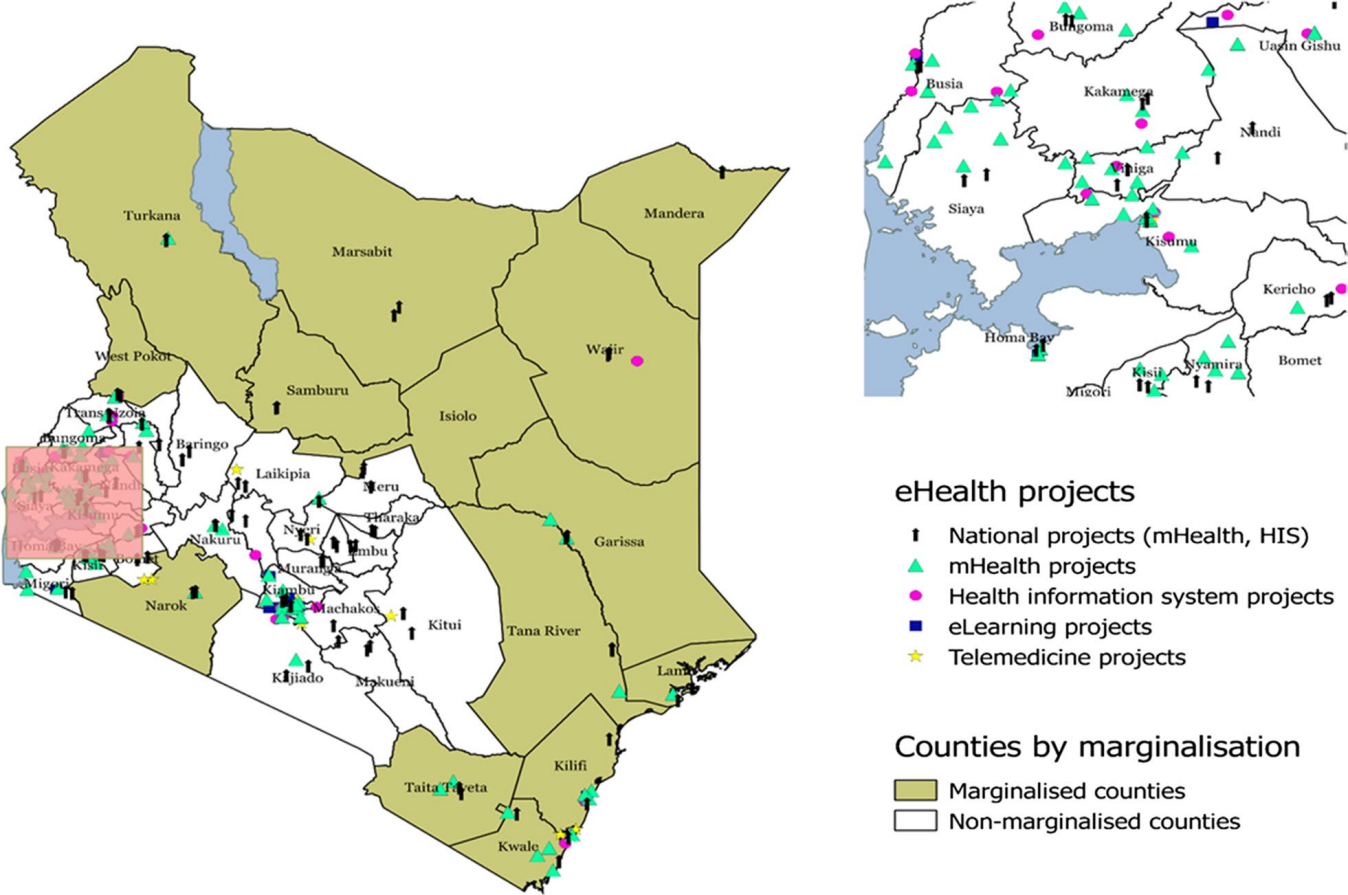
Distribution of eHealth projects in Kenya by marginalisation. Map A demonstrates eHealth projects in Kenya based on counties and categorised by strategic area of implementation and national projects. Map background is *coloured* according to county marginalisation as defined by CRA (see *colour bars*). Map B (*Inset*) *highlights* Kisumu, Vihiga, Kakamega, Siaya and neighbouring counties. *Source*: authors’ synthesis. No data permission was required

According to the Commission on Revenue Allocation (CRA), fourteen counties are classified as marginalised [26]. Of these, ten counties had at least one eHealth initiative under implementation whilst Kilifi had the highest number of projects (n = 5) with Kwale having four projects. Of the other marginalised counties, Marsabit, Samburu, Isiolo and Mandera, had no project under implementation (see Fig. 3). As shown in Fig. 3, majority of the projects (n = 128) were executed in more affluent counties.

Based on our analysis, a number of projects (n = 15) were designed to have a national reach. However, only three projects were scaled up to national level [27–31]. One of these projects relied on the innovative use of mobile money [27, 29] to provide health care and therefore it was not mapped.

## Characteristics of the projects

### Strategic area of implementation

Projects implemented in Kenya covered all eHealth strategic areas with majority (47; 69%) of them delivered through mHealth, less commonly focusing on health information systems (9; 13%), eLearning (8; 11%) and telemedicine (5; 7%) as shown in Table 2.

**Table 2:** Summary of the characteristics of eHealth projects in Kenya (Source: authors’ synthesis)

Strategic area of implementation	N = 69
mHealth	47 (69%)
Health information systems	9 (13%)
eLearning	8 (11%)
Telemedicine	5 (7%)
Thematic area	
Client education and behaviour change communication	22 (32%)
Data collection and reporting	13 (19%)
Provider training and education	6 (9%)
Financial transactions and incentives	6 (9%)
Electronic health records	5 (7%)
Provider-to-provider communication: user groups, consultation	5 (7%)
Supply chain management	4 (5%)
Other thematic areas^a^	8 (12%)
Health focus and purpose	
Primary care	19 (28%)
HIV/AIDS	17 (25%)
Maternal and child health	11 (16%)
Malaria	7 (10%)
Health financing	4 (5%)
Drug supply	4 (5%)
Other purposes^b^	7 (10%)
Evaluation status	
Not reported	41 (59%)
Feasibility and acceptability studies	9 (13%)
Randomised controlled trial	8 (12%)
Cross-sectional study	6 (9%)
Qualitative study	3 (4%)
Cohort study	1 (1%)
Non-randomised intervention study	1 (1%)

^a^The other thematic areas are sensors and point-of-care diagnostics (2; 3%), registries and vital events tracking (2; 3%), electronic decision support (2; 3%), provider work planning and scheduling (1; 1%) and human resource management (1; 1%)

^b^The other health purposes are reproductive health (2; 3%), cancer care (1; 1%), tuberculosis (1; 1%), yellow fever and rift valley fever (1; 1%), eye care (1; 1%) and epilepsy care (1; 1%)

### Thematic area

Among the 69 projects, innovations focusing on client education and behaviour change communication were the most common (22; 32%) as demonstrated in Table 2. Examples of such projects are those that use mobile SMS communication to influence patients’ adherence to medications [16, 20, 32–35] (Table 2). According to this study analysis, the second most widespread thematic area is data collection and reporting (13; 19%) involving the use of SMS and mobile applications to collate data [36–38]. Another commonly implemented eHealth innovation was electronic medical records (EMR) systems used to record and report patients data [28, 30, 39] (Table 2). The other thematic areas involved provider training and education, and financial transactions and incentives. These thematic areas were represented by six (9%) projects in each of the respective domains. Similarly, five (7%) projects were classified under electronic medical health records and provider-to-provider communication domains. Finally, three thematic areas applying sensors and point-of-care diagnostics, registries and vital events tracking, and electronic decision support were each represented by two innovations. Notably, at least one project was under implementation in each of the twelve thematic areas (Table 2).

### Health focus

In terms of health focus, this study found that nineteen (28%) projects focused on primary care followed by HIV/AIDS (17; 25%), then maternal and child health (11; 16%) and malaria (8; 11%). Twenty-three projects were designed to target multiple conditions and diseases. Majority of these (n = 19) innovations were used to collect and report health information within the primary care (19; 27%), while two projects concentrated on both HIV/AIDS and either TB or malaria. The remaining two projects were designed to address both HIV/AIDS and maternal and child health (Table 2).

### Evaluation of the projects

Out of the sixty-nine eHealth projects only 28 (41%) reported having evaluated the project under implementation. Indeed, only 8 out of the 69 projects underwent rigorous effectiveness evaluation utilising randomized controlled trials. Of the remaining 21 projects, one was non-randomized intervention studies (1%), 7 cross-sectional studies (10%), and 1 cohort study (1%), 9 were feasibility and acceptability studies (13%) while 3 (4%) were qualitative studies (Table 2). In this analysis, the non-intervention projects were analysed as cross-sectional studies or cohort studies. Another noteworthy finding is that the first eHealth project was implemented in 2001 [40–43] in Kenya while most of the projects (n = 42) were initiated in 2010 and onwards.

For the seven randomised controlled trials testing effects of SMS communication on various health outcomes had reported positive effects [16, 20, 32, 33, 44–49], while the remaining trial showed no effect of mobile alarm devices on treatment adherence for HIV patients [15]. Only one project reported a cost-effectiveness analysis of the intervention deployed [50]. This analysis showed that the use of text-message reminders is inexpensive and effective way to improve health workers adherence to malaria treatment guidelines.

Of the 15 national projects, only two reported some form of evaluation, one being a cross-sectional survey [27, 29] while the other was a qualitative study [51, 52].

## Discussion

This systematic review set out to provide an inventory of all eHealth projects in Kenya. Even though it was a challenge gathering information relating to innovations being implemented in the country, the researchers combined different methods to ensure all projects were documented and inventoried. The analyses and categorisation of the projects based on different criteria sheds light to the advancement of ICT and in particular eHealth in Kenya. According to the findings, majority of the projects were implemented and piloted in larger urban cities such as Nairobi, Kisumu and Mombasa including a few peri-urban areas (Busia, Kakamega and Vihiga). This is attributable to the fact that in major cities, as in many other African countries, there is availability of basic infrastructure such as electricity, high penetration of mobile telecommunication and network, ICT human resources and higher population literacy rates. With the increased growth of Information and Communication Technology (ICT) and mobile devices to the entire population [8, 53], the country is likely to experience escalation of eHealth solutions.

However, the growth in mobile penetration does not necessarily translate into enhanced eHealth accessibility in the entire country particularly the remote and semi-arid regions in Kenya where majority of the marginalised population reside. For example, based on this study analysis, a comparatively small number of eHealth projects are implemented in marginalised regions in Kenya where most of the vulnerable population groups reside. The marginalised areas have limited access to health care services yet they bear the highest burden of disease [54]. The major pressing health priorities in these regions are maternal and child health challenges and communicable diseases such malaria, tuberculosis, and HIV/AIDS. And although Kenya has made tremendous progress the health challenges in marginalised areas remains a significant public health concern. One of the major eHealth public health objectives is to improve access to health services through strengthening health system [3, 55] by ensuring hard-to-reach communities receive effective and quality health care. As noted by Kirigia et al., ICT treats geographically displaced resources as if they were centralized thus promoting economies of scale [3]. Therefore, embracing eHealth can indeed facilitate equitable distribution of healthcare to the marginalised areas and vulnerable population groups.

The study results shows that little effort has been put towards implementing projects in marginalised areas of Kenya to enable the population benefit from improved access to health services. Although the regions have historically experienced challenges related to poor infrastructure, unfavourable living conditions and insecurity, the devolved system of government (County governments) under implementation in Kenya as a result of the new constitution [56], has empowered each county government with resources to improve infrastructure, the socio economic status of the population including health care services among others.

The Kenya national eHealth strategy highlights the importance of the Vision 2030 [54] and the 2010 Constitution [56] key strategic areas, which are similar to the eHealth strategic areas applied in this study to analyse the projects being implemented. Out of all the listed strategic areas, mHealth interventions take precedence. Implementers favour mobile health projects due to the high geographical coverage of the mobile network particularly in developing countries [57]. The cost of setting up mobile health systems is considerably low compared to other ICT systems. Mobile Health driven initiatives provide easy accessibility to interventions effectively at any setting. The most commonly applied features are mobile text messages (SMS), software applications and multiple media interventions. Mobile health SMS communications entails a simple short message on behaviour change targeting a particular population group or a reminder clinical information message to health care providers. These types of interventions have been proven to be effective as evidenced by the small sample of evaluated mHealth interventions [16, 20, 32, 33, 45], that showed that SMS communications had positive effect on patient behaviour and health outcomes and was also cost-effective and reliable [50]. There is a possibility that many more projects would have had a positive impact if only they were evaluated. As shown by this study, about 69 eHealth projects were implemented in Kenya with only 8 showing evidence of rigorous evaluation. Additionally, only one study presented a cost-effectiveness analysis [50].

According to this study, mHealth and eHealth projects are on the rise, which could be explained by the increasing growth of ICT sector in Kenya and the political willingness to advance investment in this important industry. Indeed, according to the World Health Organization National eHealth Strategy toolkit [1], the Kenyan eHealth sector falls in the developing and building up phase [1]. In this phase, the eHealth initiatives are majorly driven by the need to improve access and quality of health care.

Another important finding in this study is that majority of the eHealth initiatives implemented in the country are funded by development agencies and international non-governmental organisations raising the issue of ownership by the Ministry of Health. Lack of ownership contributes to duplication and fragmentation of eHealth innovations under implementation. For example, based on this analysis, majority of the eHealth initiatives under implementation are fragmented and not integrated with the national health information system creating interoperability challenges. The fragmentation is due to lack of national eHealth standards and regulatory framework to guide and support eHealth innovations. Acceptability and buy-in by of the innovations by the Ministry of Health (MoH) is critical to ensure sustainability of the innovations in case it warrants scale up. As a result of this study, the relevant policy makers are in the process of developing all necessary policy documents in an effort to address some of the challenges emerging as a result of having a large and growing ICT community in Kenya involving both public and private sectors. In a few instances, the ICT sector has experienced successful public private partnerships for example business mobile products such as M-Pesa [58] shows that the scaling up of eHealth projects can be executed in Kenya with proper planning and sufficient funding.

Generally, if eHealth projects are implemented and evaluated effectively they would contribute to improving access to and quality of health care hence strengthening the overall health system in an effort to achieve good health outcomes. This would be supported by the progressive enabling environment, which is expected to grow even further once relevant policies (national eHealth and mHealth policy, regulatory framework and guiding standards) have been put in place. Despite the lack of supportive policies, eHealth has potential benefits that demand the entrenchment of eHealth initiatives in the overall Kenyan health system. However, this will only be feasible once a legal framework and guidelines have been developed by the government in partnership with key stakeholders. The government should also provide stewardship in terms of ensuring potential eHealth and mHealth projects are aligned to the health system priorities and are provided with a roadmap to guide implementation.

As illustrated by this study, projects implemented were not aligned to the MoH health needs and priorities and instead majority of projects focused on specific diseases and population groups such as HIV/AIDS and maternal and child health at primary care. Most of the eHealth projects were used to influence client education and behaviour change and to collect patient data. The popularity of these types of eHealth projects could probably be driven by the funding sources, which are mainly international development agencies or non-government organisations originating from developed countries. This is because development agencies focuses on their countries’ development assistance strategic direction while international non-governmental organisations would focus on the most popular diseases in an effort to attract funding. The sources of funding and focus on specific diseases could explain why it is costly to scale up some of these eHealth projects nationally due to lack of economies of scope. Since health systems are interconnected, it is necessary that eHealth projects be able to integrate to the overall health system. This will enhance the opportunities for a single eHealth project to be applied to more than one health issue or disease to ensure they are economical and can be scaled up to the entire population. Further research is however required to provide evidence.

### Limitations

This review has certain limitations. First, some projects may have lacked documentation or externally shared reports, probably due to the profit driven or aid funded nature of the eHealth projects whilst some projects were proprietary in nature and thus business ventures. Therefore, information from some of these types of innovations could not be shared with the authors and there might be projects that have since been implemented once the data collection had been concluded implying that this study may not be an exhaustive inventory of all eHealth innovations implemented in Kenya.

## Conclusions

This review concludes that there is a myriad of eHealth projects being implemented in urban centres rather than marginalised areas where geographical inequalities and inequities in access to health care exist in Kenya. Due to lack of government stewardship and leadership, eHealth initiatives are not aligned to the Ministry of Health priorities hence projects location is often determined by implementers. The lack of relevant policy and regulations including insufficient monitoring and evaluation to ascertain the impact or even the cost-effectiveness contributes to most projects inability to be scaled up. The implication being that the benefits arising from eHealth adoption are often not passed on to patients or beneficiaries. It is therefore imperative that government buy-in is pursued prior to the implementation of eHealth initiatives including rigorous testing coupled with cost-effectiveness and benefits analysis to ensure suitability, appropriateness, scalability and sustainability of eHealth project. This will minimise wastage of scarce economic resources and enhance the integration of the eHealth projects into the overall health systems.

## Additional file

**Additional file 1.** List of 69 eHealth projects by their characteristics in Kenya. It outlines the categorisation of the sixty-nine eHealth projects according to the geographic location (county), strategic area of implementation, project period, thematic area, health focus, description of eHealth application and evaluation status of the project under implementation.

## Abbreviations

ICT: information communication technology
WHO: World Health Organisation
EMR: electronic medical record
eHealth: electronic health
LMICs: low-and-middle income countries
MNCH: maternal and child health
IDRC: International Development Research Centre
mHealth: mobile health
elearning: electronic learning
KEMRI: Kenya Medical Research Institute
SERU: Scientific and Ethics Review Unit
SMS: Short Message Service
TB: tuberculosis
CRA: Commission on Revenue Allocation
CDI: County Development Index
MoH: Ministry of Health
