# Supplementary material for: Assessing the feasibility of eHealth and mHealth: a systematic review and analysis of initiatives implemented in Kenya
Source: BMC Res Notes. 2017 Feb 10;10:90. doi: 10.1186/s13104-017-2416-0 (PMC5301342; doi:10.1186/s13104-017-2416-0)
Supplement: Supplementary file 1 — Additional file 1. List of 69 eHealth projects by their characteristics in Kenya. It outlines the categorisation of the sixty-nine eHealth projects according to the geographic location (county), strategic area of implementation, project period, thematic area, health focus, description of eHealth application and evaluation status of the project under implementation. [file 13104_2017_2416_MOESM1_ESM.docx]

List of 69 eHealth projects by their characteristics in Kenya

This table outlines categorisation of sixty nine mapped eHealth projects according to geographic location (county), strategic area of implementation, project period, thematic area, health focus, description of eHealth application and evaluation of the application.

| **Implementation area by county** | **Project period** | **Thematic area** | **Health focus** | **Application description** | **Evaluation of applications** | **Reference** |
| --- | --- | --- | --- | --- | --- | --- |
| **mHealth** | | | | | | |
| Kisumu | 2007 – 2008 | Client education and behaviour change communication | HIV/AIDS | SMS communication used to improve adhere to ARV treatment. | RCT, Positive effect | [1] |
| Kajiado, Nairobi | 2007 – 2008 | Client education and behaviour change communication | HIV/AIDS | SMS communication used to improve adherence of ART treatment and suppress plasma HIV-1 load. | RCT, Positive effect | [2-4] |
| Nairobi | 2013 – To date | Client education and behaviour change communication | HIV/AIDS | SMS communication used to improve the retention in care among HIV-infected individuals. | RCT, Underway | [5, 6] |
| Nairobi | 2006 – 2008 | Client education and behaviour change communication | HIV/AIDS | Alarm device on adherence of HAART treatment and biological outcomes. | RCT, No effect | [7] |
| Kisumu, Homabay, Siaya, Migori, Nyamira, Kisii | 2010 – 2011 | Client education and behaviour change communication | HIV/AIDS | SMS communication on attendance of a post-operative circumcision visit. | RCT, Positive effect | [8] |
| Kiambu | 2011 – 2012 | Client education and behaviour change communication | HIV/AIDS | Use of SMS communications to measure sexual behaviour and PreP adherence. | RCT, Positive use | [9] |
| Kisii, Kwale | 2009 – 2010 | Provider work planning and scheduling | Malaria | SMS communication on correct management of artemether-lumefantrine for paediatric use. | RCT, Positive effect | [10-12] |
| Siaya | 2013 – To date | Client education and behaviour change communication | Malaria | SMS communication used to improve patients’ adherence to malaria treatment and a day 3 post treatment review. | RCT, Underway  Cross-sectional pre-intervention survey conducted. | [13, 14] |
| Busia, Kakamega, Bungoma | 2009 – 2009 | Registries and vital events tracking | Maternal & child health | SMS communication and voice calls used for subject enrolment and data collection. | Prospective cohort study, Positive effect. | [15] |
| Homabay | 2013 | Client education and behaviour change communication | HIV/AIDS | Acceptability of mobile phone communications and content in support of PMTCT. | Qualitative study. | [16] |
| Meru | 2011 – 2011 | Provider training and education | Maternal health | Non-interactive video training compared against hands-on training for post-partum management training. | Controlled non randomised intervention study, No significant difference. | [17] |
| Machakos, Nyamira, Vihiga, Kwale, Garissa | 2011 – 2012 | Data collection and reporting | Malaria | SMS used for reporting of malaria commodities and disease surveillance to reduce stock outs | Prospective longitudinal study. | [18, 19] |
| National | 2008 – 2010 | Financial transactions and incentives | Health financing | Mobile money use by households to respond to adverse health events. | Cross-sectional survey, Positive effect. | [20-22] |
| Nairobi, Kajiado | 2010 – 2011 | Client education and behaviour change communication | Reproductive health | SMS communications used to provide basic information on the different contraceptive methods. | Post-intervention acceptability survey. Effective method. | [23, 24] |
| Siaya | 2011-2011 | Financial transactions and incentives | Child health | SMS reminder & conditional cash transfer used to improve timely immunization | Feasibility trial. Potentially useful. | [25] |
| Nairobi, Kisumu, Busia | 2012 –2012 | Data Collection and reporting | Child health | Mobile App used to collect immunization data. | Cross-sectional study. | [26, 27] |
| National | 2011 – To date | Client education and behaviour change communication | Primary care | Mobile App used to provide health information to patients on request. | Not reported | [28, 29] |
| Nairobi | 2009- To date | Financial transactions and incentives | Maternal health | Mobile Money transfers used to enable fistula repair surgery. | Not reported | [30] |
| Nairobi | 2012 – 2012 | Client education and behaviour change communication | Child health | SMS communication on timely immunization | Not reported | [31] |
| National | 2006 – To date | Client education and behaviour change communication | HIV/AIDS | Mobile phone games aimed to improve patient HIV awareness. | Not reported | [32, 33] |
| National | 2006 – To date | Client education and behaviour change communication | HIV/AIDS | Toll free number that provides HIV information and counselling. | Not reported | [34, 35] |
| National | 2014 – To date | Client education and behaviour change communication | Epilepsy Care | Toll free number used to provide information on epilepsy. | Not reported | [36] |
| Nairobi | 2008 – 2009 | Client education and behaviour change communication | Tuberculosis | Mobile video recordings used to improve direct observation of treatment of TB. | Feasibility assessment. Viable option. | [37, 38] |
| National | 2012 – To date | Client education and behaviour change communication | Primary care | Web portal and SMS platform used to provide health information to users on request. | Not reported | [39] |
| Busia, Bungoma, Kakamega, Vihiga, Kisumu | 2009 – 2011 | Electronic health records | HIV/AIDS, TB, Malaria | Mobile devices are used to collect household data | Not reported | [40-42] |
| National | 2009 – To date | Supply chain management | Drug supply | SMS communications used to verify the genuineness of drugs. | Not reported | [43] |
| National | 2010 – To date | Supply chain management | Drug supply | SMS communication used to verify the genuineness of drugs. | Not reported | [44] |
| Nairobi | 2011 – To date | Electronic health record | HIV/AIDS | Mobile app used to upload HIV test data to a central database. | Not reported | [45] |
| Kiambu | 2014 – To date | Electronic decision support: information, protocols, algorithms, checklists | Primary care | Mobile app used to assist community health workers to make diagnostic decisions. | Not reported | [46] |
| Kilifi, Busia, Kwale | 2011 – To date | Electronic health record | Maternal & child health | Mobile app used to collect information on the mother during pregnancy and the child born. | Not reported | [47] |
| Siaya | 2009 – To date | Registries and vital events tracking | Maternal & child health | SMS communication used to collect data on the mother and children during routine check-ups. | Feasibility assessment. | [48, 49] |
| Nairobi | 2014 – To date | Financial transactions and incentives | Health financing | Mobile App and USSD code that is used to calculate health insurance premiums and purchase the selected insurance package. | Not reported | [50, 51] |
| National | 2008 – To date | Financial transactions and incentives | Health financing | Online mobile application is used to provide health insurance linked to the client’s m-pesa account. | Not reported | [52] |
| National | 2009 – 2014 | Human resource management | Primary care | Mobile devices and information technology software used to manage health workers. | Qualitative study | [53-55] |
| Kisumu, Homabay, Siaya, Migori, Nyamira, Kisii | 2006 – To date | Provider-to-provider communication: user groups, consultation | HIV/AIDS | Toll free number is used to provide HIV related information to health workers. | Cross-sectional survey | [56, 57] |
| Nairobi, Mombasa, Kwale, Kilifi, Tana River, Lamu, Taita-Taveta | 2012 – To date | Electronic decision support: information, protocols, algorithms, checklists | HIV/AIDS, Maternal & child health | A decision and support tool based on the CommCare mobile software platform. | Not reported | [58] |
| Narok | 2011 – To date | Data Collection and Reporting | Primary care | Mobile software that is used for data capture and reporting. | Not reported | [59, 60] |
| Nairobi | 2014- To date | Sensors and point-of-care diagnostics. | Malaria | Mobile phones connected to external mobile devices are used to perform diagnostic tests. | Not reported | [61] |
| Nairobi, Kiambu, Uasin Gishu, Bungoma | 2011 – To date | Client education and behaviour change communication | Maternal health | Voice communication used to screen pregnant women and new mothers and make referrals. | Not reported | [62, 63] |
| Garissa | 2010 – 2010 | Data collection and reporting | Malaria | Android Application used to collect data. | Not reported | [64] |
| Kakamega, Vihiga, Bungoma, Busia, Uasin Gishu, Kisumu, West Pokot, Trans-Nzoia, Turkana | 2008 – To date | Data collection and reporting | Primary care | Open Source mobile application used to collect data. | Cross-sectional survey. System was useful. | [65, 66] |
| Siaya, Migori,Nyamira, Kisii, Homabay, Kisumu, Kakamega, Vihiga, Bungoma, Busia, Trans-Nzoia, Nakuru, Kericho, Nandi, Uasin Gishu | 2008 –2011 | Data collection and reporting | Reproductive health | SMS used to collect and report patient data. | Qualitative study | [67] |
| Busia, Nakuru, Machakos, Kilifi, Taita Taveta | 2008 – 2013 | Client education and behaviour change communication | HIV/AIDS | SMS communication used to provide information to targeted audience. | Not reported | [68, 69] |
| Nairobi | 2010- to date | Electronic Health Record | Maternal & child health. | SMS and Web application used to register vital events and disease surveillance. | Not reported | [70] |
| Kisumu | 2013- To date | Data collection and reporting | Malaria | A mobile device, called a Deki reader is used to diagnose malaria and transmit the results. | Feasibility study | [71, 72] |
| Kajiado, Busia | 2012- To date | Data collection and reporting | Yellow fever & Rift Valley fever | A mobile SMS-based disease outbreak alert system. | Not reported | [73] |
| National | 2009-To date | Supply chain management | Drug supply | Mobile application used procure medical supplies. | Not reported | [74] |
| **Health Information Systems** | | | | | | |
| Kakamega, Vihiga, Bungoma, Busia, Uasin Gishu, Kisumu, West Pokot, Trans-Nzoia | 2001 – To date | Electronic health records | Primary care | Electronic medical record used to collect and store patient data. | Feasibility study, Data quality improved. | [75, 76, 66, 77-80] |
| National | 2010 – To date | Data collection and reporting | Primary care | Health information system used to collect aggregated patient data from health centres and report it to the national level. | Not reported | [81, 82] |
| Nairobi | 2012 – To date | Financial transactions and incentives | Health financing | EMR that connects health insurance providers and health centres for easy processing of health claims. | Not reported | [83] |
| Nairobi, Wajir, Kilifi, Machakos, Trans-Nzoia, Busia | 2011 – To date | Data collection and reporting | Primary care | Web based system used to collect and share surveillance information from public health laboratories. | Not reported | [84, 85] |
| Nairobi, Kajiado, Nakuru | 2005 – To date | Data collection and reporting | Primary care | EMR used to collect and maintain patient records concerning gender violence. | Not reported | [86] |
| Nairobi, Mombasa, Uasin Gishu, Kericho, Busia, Kisumu | 2012 – To date | Data collection and reporting | HIV/AIDS, Child health | EMR used to record and communicate HIV test data to health centres through the internet and SMS communication. | Not reported | [87-90] |
| Kajiado | 2011 –2012 | Provider-to-provider communication: user groups, consultation | Primary care | EMR used to relay health information to health workers. | Not reported | [91, 92] |
| National | 2013- To date | Supply chain management | Drug supply | Web based tool used for commodity management. | Not reported | [93, 94] |
| Kisumu | 2013- To date | Data collection and reporting | Maternal & child health | EMR used to collect and maintain patient records concerning maternal and child health. | Not reported | [95] |
| **elearning** | | | | | | |
| Kilifi | 2002 – 2002 | Provider training and education | Primary care | PDA platform used to provide medical information to health workers. | Cross-sectional survey | [96] |
| Nairobi, Uasin Gishu | 2011 – 2011 | Client education and behaviour change communication | Cancer care | Mobile phone and internet use for cervical cancer information by patients in a hospital setting. | Cross-sectional feasibility study | [97] |
| Kiambu | 2006 – To date | Provider training and education | Primary care | Web based and PDA platforms used to provide medical information to health workers. | Not reported | [98] |
| Nairobi | 2014 – To date | Provider-to-provider communication: user groups, consultation | Eye Care | Web portal used to link eye health professions for further consultation. | Not reported | [99] |
| Migori, Kiambu | 2011 – To date | Provider training and education | Primary care | PDA platform used to provide medical information and diagnostic support for health workers. | Not reported | [100, 101] |
| Nairobi, Kiambu, Busia | 2007 – To date | Provider training and education | HIV/AIDS, TB | Web portal used to provide information to health workers. | Not reported | [102] |
| Homabay | 2007 – To date | Client education and behaviour change communication | HIV/AIDS | Internet provided on site as an incentive to do HIV test and learn about HIV. | Not reported | [103] |
| National | 2005 – 2010 | Provider training and education | Primary care | An e-learning platform used to train nurses. | Not reported | [104] |
| **Telemedicine** | | | | | | |
| Nairobi, Kisumu, Mombasa | 2010 – 2010 | Provider-to-provider communication: user groups, consultation | Cancer care | Video application used for histological analysis by the health care workers. | Feasibility study | [105] |
| Kajiado, Nyeri | 2010 – To date | Provider-to-provider communication: user groups, consultation | Primary care | Web App and mobile calls used by low cadre health workers for consultation with high cadre health workers. | Concordance & feasibility study | [106-108] |
| National | 2012 – 2014 | Client education and behaviour change communication | Primary care | Telephone call used for triage of patients before being referred to a health centre. | Not reported | [109, 110, 29] |
| Nairobi | 2010 – To date | Client education and behaviour change communication | Primary care | Telephone call used for triage of patients before being referred to a health centre. | Not reported | [111] |
| Narok, Bomet, Kilifi, Kitui, Laikipia, Mombasa | 2011 – 2012 | Sensors and point-of-care diagnostics | Primary care | Proprietary medical devices are used for medical diagnosis and consultation. | Not reported | [112, 113] |

# **References**

1. Pop-Eleches C, Thirumurthy H, Habyarimana J, Graff Zivin J, Goldstein M, De Walque D et al. Mobile phone technologies improve adherence to antiretroviral treatment in resource-limited settings: a randomized controlled trial of text message reminders. Aids. 2011;25:825-34.

2. Lester RT, Mills EJ, Kariri A, Ritvo P, Chung M, Jack W et al. The HAART cell phone adherence trial (WelTel Kenya1): a randomized controlled trial protocol. Trials. 2009;10(1):87.

3. Lester RT, Ritvo P, Mills EJ, Kariri A, Karanja S, Chung MH et al. Effects of a mobile phone short message service on antiretroviral treatment adherence in Kenya (WelTel Kenya1): a randomised trial. The Lancet. 2010;376(9755):1838-45.

4. Van Der Kop ML, Karanja S, Thabane L, Marra C, Chung MH, Gelmon L et al. In-depth analysis of patient-clinician cell phone communication during the WelTel Kenya1 antiretroviral adherence trial. PloS one. 2012;7(9):e46033.

5. van der Kop ML, Ojakaa DI, Patel A, Thabane L, Kinagwi K, EkstrÃ¶m AM et al. The effect of weekly short message service communication on patient retention in care in the first year after HIV diagnosis: study protocol for a randomised controlled trial (WelTel Retain). BMJ open. 2013;3(6).

6. Weltel International mHealth Society. Projects. 2014. <http://www.weltel.org/projects/>. Accessed 20th May 2014.

7. Chung MH, Richardson BA, Tapia K, Benki-Nugent S, Kiarie JN, Simoni JM et al. A randomized controlled trial comparing the effects of counseling and alarm device on HAART adherence and virologic outcomes. PLoS medicine. 2011;8(3):e1000422.

8. Odeny TA, Bailey RC, Bukusi EA, Simoni JM, Tapia KA, Yuhas K et al. Text messaging to improve attendance at post-operative clinic visits after adult male circumcision for HIV prevention: a randomized controlled trial. PloS one. 2012;7(9):e43832.

9. Curran K, Mugo NR, Kurth A, Ngure K, Heffron R, Donnell D et al. Daily Short Message Service Surveys to Measure Sexual Behavior and Pre-exposure Prophylaxis Use Among Kenyan Men and Women. AIDS and Behavior. 2013;17(9):2977-85.

10. Jones CO, Wasunna B, Sudoi R, Githinji S, Snow RW, Zurovac D. "Even if you know everything you can forget": health worker perceptions of mobile phone text-messaging to improve malaria case-management in Kenya. PloS one. 2012;7(6):e38636.

11. Zurovac D, Larson BA, Sudoi RK, Snow RW. Costs and Cost-Effectiveness of a Mobile Phone Text-Message Reminder Programmes to Improve Health Workers' Adherence to Malaria Guidelines in Kenya. PloS one. 2012;7(12):e52045.

12. Zurovac D, Sudoi RK, Akhwale WS, Ndiritu M, Hamer DH, Rowe AK et al. The effect of mobile phone text-message reminders on Kenyan health workers' adherence to malaria treatment guidelines: a cluster randomised trial. The Lancet. 2011;378(9793):795-803.

13. Talisuna A, Zurovac D, Githinji S, Oburu A, Malinga J. Efficacy of Mobile Phone Short Message Service (SMS) Reminders on Malaria Treatment Adherence and Day 3 Post-Treatment Reviews (SMS-RES-MAL) in Kenya: A Study Protocol. J Clin Trials. 2015;5(217):2167-0870.1000217.

14. Otieno G, Githinji S, Jones C, Snow RW, Talisuna A, Zurovac D. The feasibility, patterns of use and acceptability of using mobile phone text-messaging to improve treatment adherence and post-treatment review of children with uncomplicated malaria in western Kenya. Malaria journal. 2014;13(1):44.

15. Gisore P, Shipala E, Otieno K, Rono B, Marete I, Tenge C et al. Community based weighing of newborns and use of mobile phones by village elders in rural settings in Kenya: a decentralised approach to health care provision. BMC pregnancy and childbirth. 2012;12(1):15.

16. Jennings L, Ong J, Simiyu R, Sirengo M, Kassaye S. Exploring the use of mobile phone technology for the enhancement of the prevention of mother-to-child transmission of HIV program in Nyanza, Kenya: a qualitative study. BMC public health. 2013;13(1):1131.

17. Nilsson C, SÃ¸rensen BL, SÃ¸rensen JL. Comparing hands-on and video training for postpartum hemorrhage management. Acta obstetricia et gynecologica Scandinavica. 2014;93(5):517-20.

18. Githinji S, Kigen S, Memusi D, Nyandigisi A, Mbithi AM, Wamari A et al. Reducing stock-outs of life saving malaria commodities using mobile phone text-messaging: SMS for Life study in Kenya. PloS one. 2013;8(1):e54066.

19. Githinji S, Kigen S, Memusi D, Nyandigisi A, Wamari A, Muturi A et al. Using mobile phone text messaging for malaria surveillance in rural Kenya. Malaria journal. 2014;13(1):107.

20. Suri T, Jack W, Stoker TM. Documenting the birth of a financial economy. Proceedings of the National Academy of Sciences. 2012;109(26):10257-62.

21. Maurer B. Mobile money: Communication, consumption and change in the payments space. Journal of Development Studies. 2012;48(5):589-604.

22. Mas I, Radcliffe D. Mobile payments go viral: M-PESA in Kenya. 2010.

23. FHI 360. Mobile for Reproductive Health Project: Kenya. 2012. <http://m4rh.fhi360.org/wp-content/uploads/2013/03/m4RH-Summary-Kenya.pdf>. Accessed 20th May 2014.

24. Vahdat HL, Lâ€™Engle KL, Plourde KF, Magaria L, Olawo A. There are some questions you may not ask in a clinic: Providing contraception information to young people in Kenya using SMS. International Journal of Gynecology & Obstetrics. 2013;123:e2-e6.

25. Wakadha H, Chandir S, Were EV, Rubin A, Obor D, Levine OS et al. The feasibility of using mobile-phone based SMS reminders and conditional cash transfers to improve timely immunization in rural Kenya. Vaccine. 2013;31(6):987-93.

26. Mbabazi WB, Tabu CW, Chemirmir C, Kisia J, Ali N, Corkum MG et al. Innovations in communication technologies for measles supplemental immunization activities: lessons from Kenya measles vaccination campaign, November 2012. Health Policy Plan. 2014. doi:czu042 [pii]

10.1093/heapol/czu042 [doi].

27. Datadyne. Magpi: Fast, Easy Mobile Data for Health, Agriculture, Education, Conservation, Commerce and More! 2014. <http://www.datadyne.org/magpi-mobile/>. Accessed 20th May 2014.

28. Shimba Technologies. medAfrica. 2012. <http://www.medafrica.org/index.php>. Accessed 20th May 2014.

29. Talbot D. Kenya's Mobile Prescription. Technology Review. 2012; p. 8.

30. Health Unbound, PHImHealth. m-Money For Women with Fistula. 2012. <http://www.healthunbound.org/node/1995>. Accessed 20th May 2014.

31. Fentress K. M-chanjo: Saving lives by mixing health care with mobile technology. 2012. <http://www.urb.im/nr/120729mc>. Accessed 20th May 2014.

32. ZMQ. Freedom HIV/AIDS. 2007. <http://www.freedomhivaids.in/FreedomHivAids.htm>. Accessed 20th May 2014.

33. Noordam C. Using Mobile Phones to Strengthen Health Systems, with a Focus on Maternal and Newborn Health. Royal tropical institute (KIT); 2009.

34. Liverpool VCT Care & Treatment. one2one. 2012. <http://www.one2onekenya.org/site/>. Accessed 20th May 2014.

35. LVCT. Lvct Health. 2011. <http://www.lvct.org/>. Accessed 20th May 2014.

36. Safaricom. Fafanuka Dial *215# - Epilepsy Care Service. 2014. <https://www.youtube.com/watch?v=2RDyWTHdw6s>. Accessed 10th July 2014.

37. Danya International. Mobile Direct Observation Treatment (MDOT) of Tuberculosis Patients Pilot Feasibility Study in Nairobi, Kenya. Nairobi. 2009. <http://www.danya.com/files/mdot%20final%20report.pdf>. Accessed 20th May 2014.

38. Hoffman JA, Cunningham JR, Suleh AJ, Sundsmo A, Dekker D, Vago F et al. Mobile direct observation treatment for tuberculosis patients: a technical feasibility pilot using mobile phones in Nairobi, Kenya. American journal of preventive medicine. 2010;39(1):78-80.

39. Avallain Africa. What is iAfya? 2014. <http://www.iafya.org/help/>. Accessed 20th May 2014.

40. IFPMA. HIV/AIDS - Healthcare Capacity: Health at Home/Kenya. 2012. <http://partnerships.ifpma.org/partnership/hiv-aids-healthcare-capacity-health-at-home-kenya>. Accessed 20th May 2014.

41. Innovations CfHM. Health at Home/Kenya. 2015. <http://healthmarketinnovations.org/program/health-homekenya>. Accessed 16th July 2015.

42. Office of U.S. Global AIDS Coordinator and the Bureau of Public Affairs USSD. Kenya: Bringing Home-based HIV Counseling and Testing to Western Kenya (May 2009). 2009. <http://www.pepfar.gov/press/docs/124853.htm>. Accessed 20th May 2014.

43. Sproxil. We began in response to a serious global problem. 2014. <http://sproxil.com/about-us.html>. Accessed 19th May 2014.

44. mPedigree Network. We Are the mPedigree Network. 2014. <http://mpedigree.net/mpedigreenet/index.php/about-us/overview-the-mpedigree-network>. Accessed 19th May 2014.

45. Paige. How the Mobile Health Platform gives hope to HIV-positive babies for a normal life. 2011. <http://nethope.org/blog/2011/12/how-the-mobile-health-platform-gives-hope-to-hiv-positive-babies-for-a-norm/>. Accessed 20th May 2014.

46. Sana. Sana Technology Platform. 2014. <http://sana.mit.edu/platform/>. Accessed 20th May 2014.

47. World Vision International. mHealth: Kenya. 2014. <http://www.wvi.org/health/mhealth-kenya>. Accessed 20th May 2014.

48. Berg M, Wariero J, Modi V. Every child counts: The use of SMS in Kenya to support the community based management of acute malnutrition and malaria in children under five. Columbia university. Earth institute. ChildCount: with UNICEF Innovation group; 2009.

49. Childcount+. ChildCount+, a Community Health Events Reporting and Binary Options Brokers Alerts System. 2009. <http://www.childcount.org/>. Accessed January 22 2015.

50. UAP Insuarance. AfyaImara. UAP Insuarance, Nairobi. 2014. <http://myuap.uap-group.com/afyaLab/uap/index.php/pg/general>. Accessed 19th May 2014.

51. UAP Insuarance Staff Writer. UAP insurance launches mobile app. CIO, Nairobi. 2014. <http://www.cio.co.ke/news/top-stories/uap-insurance-launches-mobile-app>. Accessed 19 May 2014.

52. Changamka Micro Health Limited. Who we are. Nairobi. 2014. <http://changamka.co.ke/about-us.html>. Accessed 19th May 2014.

53. IntraHealth International. Capacity Kenya. 2009. <http://www.intrahealth.org/page/capacity-kenya>. Accessed 19th May 2014.

54. IntraHealth International. Kenya. Nairobi. 2014. <http://www.intrahealth.org/files/media/kenya/Country_Brief_Kenya_web%20ready.pdf>.

55. The Capacity Project. Planning, Developing and Supporting the Health Workforce: The Capacity Project2010.

56. Faces Kenya. Uliza. 2014. <http://www.faces-kenya.org/what-we-do/uliza/>. Accessed 20th May 2014.

57. Karari C, Tittle R, Penner J, Kulzer J, Bukusi EA, Marima R et al. Evaluating the uptake, acceptability, and effectiveness of Uliza! clinicians' HIV hotline: a telephone consultation service in Kenya. Telemedicine and e-Health. 2011;17(6):420-6.

58. Pathfinder International. mHMtaani: Bringing Mobile Health to the Community. 2014. <http://www.pathfinder.org/our-work/projects/mobile-phones-for-maternal-and-child-health.html>. Accessed 19th May 2014.

59. Vecna Cares. CliniPAK TC. 2014. <http://www.vecnacares.org/solutions/clinipak/>. Accessed 19th May 2014.

60. Vecna Technoligies I. Vecna Cares Upgrages CliniPAK Units in Kenya. 2013. <http://www.vecna.com/vecna-cares-upgrades-clinipak-units-in-kenya>. Accessed 28th January 2015.

61. Mobile Diagnostic Services. Innovative Solutions. 2014. <http://www.modise.org/>. Accessed 19th May 2014.

62. Baby Monitor. How it works. 2011. <http://www.babymonitor.co/>. Accessed 20th May 2014.

63. InSTEDD. Baby Monitor: Interactive Voice Response System for Clinical Screening of Pregnant Women. 2011. <http://instedd.org/baby-monitor/>. Accessed 10th July 2014.

64. Hoibak S, Schilperoord M. Android Phones for Mosquito Net Surveys. Forced Migration Review. 2011(Issue 38):30-3.

65. Open Data Kit (ODK). About. 2008. <http://opendatakit.org/about/>. Accessed 20th May 2014.

66. Rajput ZA, Mbugua S, Amadi D, ChepnÇµeno V, Saleem JJ, Anokwa Y et al. Evaluation of an Android-based mHealth system for population surveillance in developing countries. Journal of the American Medical Informatics Association. 2012;19(4):655-9.

67. Judy G, ANDREWS H, APPLEFORD G, RAMANANTSOA B, HANITRINIAINA O, DEIPARINE R et al. Using mobile phone text messages (SMS) to collect health service data: Lessons from social franchises in Kenya, Madagascar and the Philippines. Journal of Health Informatics in Developing Countries. 2012;6(2).

68. FHI 360. ROADS to a Healthy Future (ROADS II) in Kenya. 2013. <http://www.fhi360.org/projects/roads-healthy-future-roads-ii-kenya>. Accessed 20th May 2014.

69. FHI 360. Roads II Countries: Kenya. 2008. <http://www.fhi360.org/sites/default/files/media/documents/ROADS%20II%20Kenya.pdf>. Accessed 11th July 2014.

70. Medic Mobile. We are all health workers. 2005. <http://medicmobile.org/>. Accessed February 5th 2015.

71. Fio Corporation. Fionet in the Field: KEMRI Research Project 2014. <https://www.youtube.com/watch?v=t_vrl2t7ASM>. Accessed February 5th 2015.

72. Ministry of Health Kenya. KDOMC Fionet Pilot Report; The Kenya Division of Malaria Control Experience in the Deployment of Fionet to Improve Diagnosis, Data Capture and Programme Management in Kisumu, Kenya: Ministry of Health Kenya, 2013.

73. Disease Surveillance and Response Unit - Ministry of Health. Mobile SMS Based Disease Outbreak Alert System. 2015. <http://ddsr.or.ke/mSOS/about>. Accessed 10th March 2015 2015.

74. KEMSA. KEMSA E-mobile. 2015. <http://kemsa.co.ke/index.php?option=com_content&view=article&id=66&Itemid=153>. Accessed 10th March 2015 2015.

75. Vanderbilt University. The Moi Schools of Medicine & Public Health, Kenya. Eldoret. 2001. <http://www.vecd.org/trainee-sites/the-moi-schools-of-medicine-public-health-kenya/>. Accessed 20th May 2014.

76. Monda J, Keipeer J, Were MC. Data integrity module for data quality assurance within an e-health system in sub-Saharan Africa. Telemedicine and e-Health. 2012;18(1):5-10.

77. Fazen LE, Chemwolo BT, Songok JJ, Ruhl LJ, Kipkoech C, Green JM et al. AccessMRS: Integrating OpenMRS with Smart Forms on Android. Studies in health technology and informatics. 2012;192:866-70.

78. Were MC, Kariuki J, Chepng'eno V, Wandabwa M, Ndege S, Braitstein P et al. Leapfrogging paper-based records using handheld technology: experience from Western Kenya. Studies in health technology and informatics. 2009;160(Pt 1):525-9.

79. Diero L, Rotich JK, Bii J, Mamlin BW, Einterz RM, Kalamai IZ et al. A computer-based medical record system and personal digital assistants to assess and follow patients with respiratory tract infections visiting a rural Kenyan health centre. BMC medical informatics and decision making. 2006;6(1):21.

80. Haskew J, Rø G, Saito K, Turner K, Odhiambo G, Wamae A et al. Implementation of a cloud-based electronic medical record for maternal and child health in rural Kenya. International Journal of Medical Informatics. 2015.

81. Afyainfo. The AfyaInfo Project. 2014. <http://www.afyainfo.org/index.php/features/the-afyainfo-project>. Accessed 20th May 2014 2014.

82. Sahay S, SÃ¦bÃ¸ J, Braa Jr. Scaling of HIS in a global context: Same, same, but different. Information and Organization. 2013;23(4):294-323.

83. Savannah Informatics Limited. Welcome to Savannah Informatics. 2014. <http://www.savannahinformatics.com/index.php>. Accessed 20th May 2014.

84. East African Community - Health. E.A Public Health Laboratory Networking Project. 2014. <http://www.eac.int/health/>. Accessed 20th May 2014.

85. Ope M, Sonoiya S, Kariuki J, Mboera LE, Gandham RN, Schneidman M et al. Regional initiatives in support of surveillance in East Africa: the East Africa integrated disease surveillance network (EAIDSNet) experience. Emerging health threats journal. 2013;6.

86. Gender Violence Recovery Centre. Gender Violence Monitoring Unit. Nairobi. 2014. <http://www.gvrc.or.ke/index.php/programs/gvmu>. Accessed 20th May 2014.

87. National AIDS/STD Control Programme (NASCOP). National EID Dashboard. 2015. <http://www.nascop.org/eid/overall.php>. Accessed 4th February 2015.

88. Clinton Health Access Initiative. Scaled use of Cloud, Mobile and Internet Technology in Public Health Diagnostics/Laboratories. 2013. <http://www.aphl.org/conferences/proceedings/Documents/2013/2013-APHL-Annual-Meeting/23Hungu.pdf>. Accessed 3rd June 2013.

89. Mark Okutah. HP opens lab in Strathmore varsity for healthcare solutions Business Daily. 2014 June 11, 2014.

90. Rochman E, Vogel A. Improving testing and treatment for babies exposed to HIV. ITU News. 2013.

91. European Space Agency. Sahel - Satellite African e-HEalth Validation 2011. <http://artes-apps.esa.int/projects/sahel>. Accessed 20th May 2014.

92. Inigo P. Satellite African eHealth validation. 2012. <http://artes-apps.esa.int/sites/default/files/5.4-SAHEL-PP-053-CASA-PI-ESA-Artes-workshop-Harwell.pdf>. Accessed 11th June 2014.

93. Ciuri S. Strathmore whizz kids offer medical supplies solution. Business Daily. 2013.

94. Ministry of Health Kenya. Health commodities management platform (HCMP); facility level user guide. 2013.

95. MicroClinic Technologies Ltd K. Zidi Health Innovation. 2013. <http://www.microclinictech.com/index.php/zidi-microclinic-technologies-innovation>. Accessed 10th March 2015 2015.

96. Merrell RC, Merriam N, Doarn C. Information support for the ambulant health worker. Telemedicine Journal & e-Health. 2004;10(4):432-6.

97. Kivuti-Bitok LW, McDonnell G, Pokhariyal GP, Roudsari AV. Self-reported use of internet by cervical cancer clients in two National Referral Hospitals in Kenya. BMC research notes. 2012;5(1):559.

98. Cisco Internet Business Solutions Group. Rural Kenya Adopts Wireless Technology and Unique Medical Map to Improve Patient Care and Student Education. 2006. <http://www.cisco.com/web/about/ac79/docs/wp/Kijabe_Hospital_CS_1009a.pdf>. Accessed 20th May 2014.

99. Project Orbis International Inc. Cyber-Sight. 2013. <http://www.cybersight.org/bins/home.asp>. Accessed 20th May 2014.

100. Health eVillages. About. 2014. <http://www.healthevillages.org/about/>. Accessed 19th May 2014.

101. Linder M. Mobile Health Technology: Touching Lives Across the Globe. Health Management Technology. 2012 June 2012.

102. Health [e] Foundation. What we do. 2014. <http://www.healthefoundation.eu/get/7100/1/home>. Accessed 19th May 2014.

103. Organic Health Response. Ekialo Kiona Club: “Cyber-VCT”. 2014. <http://organichealthresponse.org/programs/solidarity/cyber-vct/>. Accessed 20th May 2014.

104. Amref. eHealth. 2005. <http://amref.org/what-we-do/capacity-building/ehealth-support-unit/>. Accessed 19th May 2014.

105. Kumar N, Busarla SVP, Sayed S, Kirimi JM, Okiro P, Gakinya SM et al. Telecytology in East Africa: a feasibility study of forty cases using a static imaging system. Journal of telemedicine and telecare. 2012;18(1):7-12.

106. Qin R, Dzombak R, Amin R, Mehta K. Reliability of a telemedicine system designed for rural Kenya. Journal of primary care & community health. 2013;4(3):177-81.

107. Smith SE, Ludwig JT, Chinchilli VM, Mehta K, Stoute JA. Use of Telemedicine to Diagnose Tinea in Kenyan Schoolchildren. Telemedicine and e-Health. 2013;19(3):166-8.

108. Mashavu Networked Health Solutions. Mashavu: Implementing Change. 2014. <http://mashavukenya.wordpress.com/>. Accessed 20th May 2014.

109. SquadLab. Safaricom - Daktari 1525 2012. <https://www.youtube.com/watch?v=x6anx66G_2w>. Accessed 11th July 2014.

110. Safaricom. Social Innovation. 2012. <http://www.safaricom.co.ke/personal/value-added-services/social-innovation>. Accessed 20th March 2014.

111. m-Care Medical Centre. Introduction. 2014. <http://www.m-care.co.ke/content/introduction.html>. Accessed 20th May 2014.

112. AMD Global Telemedicine. Our Telemedicine Products 2014. <http://www.amdtelemedicine.com/telemedicine-equipment/>. Accessed 20th May 2014 2014.

113. Paul Chester Children's Hope Foundation (PCCHF). What we do. 2014. <http://www.pcchf.org/about.html>. Accessed 20th May 2014.
